# Serum biomarkers reflecting specific tumor tissue remodeling processes are valuable diagnostic tools for lung cancer

**DOI:** 10.1002/cam4.303

**Published:** 2014-07-18

**Authors:** Nicholas Willumsen, Cecilie L Bager, Diana J Leeming, Victoria Smith, Claus Christiansen, Morten A Karsdal, David Dornan, Anne-Christine Bay-Jensen

**Affiliations:** 1 Biomarkers & Research, Nordic Bioscience A/SDK-2730, Herlev, Denmark; 2 Biology, Gilead Sciences Inc.Foster City, California, 94404

**Keywords:** Extracellular matrix, lung cancer, protein fingerprint, remodeling, serum biomarkers, tumor tissue

## Abstract

Extracellular matrix (ECM) proteins, such as collagen type I and elastin, and intermediate filament (IMF) proteins, such as vimentin are modified and dysregulated as part of the malignant changes leading to disruption of tissue homeostasis. Noninvasive biomarkers that reflect such changes may have a great potential for cancer. Levels of matrix metalloproteinase (MMP) generated fragments of type I collagen (C1M), of elastin (ELM), and of citrullinated vimentin (VICM) were measured in serum from patients with lung cancer (*n* = 40), gastrointestinal cancer (*n* = 25), prostate cancer (*n* = 14), malignant melanoma (*n* = 7), chronic obstructive pulmonary disease (COPD) (*n* = 13), and idiopathic pulmonary fibrosis (IPF) (*n* = 10), as well as in age-matched controls (*n* = 33). The area under the receiver operating characteristics (AUROC) was calculated and a diagnostic decision tree generated from specific cutoff values. C1M and VICM were significantly elevated in lung cancer patients as compared with healthy controls (AUROC = 0.98, *P* < 0.0001) and other cancers (AUROC = 0.83 *P* < 0.0001). A trend was detected when comparing lung cancer with COPD+IPF. No difference could be seen for ELM. Interestingly, C1M and VICM were able to identify patients with lung cancer with a positive predictive value of 0.9 and an odds ratio of 40 (95% CI = 8.7–186, *P* < 0.0001). Biomarkers specifically reflecting degradation of collagen type I and citrullinated vimentin are applicable for lung cancer patients. Our data indicate that biomarkers reflecting ECM and IMF protein dysregulation are highly applicable in the lung cancer setting. We speculate that these markers may aid in diagnosing and characterizing patients with lung cancer.

## Introduction

Lung cancer is a significant health issue and the leading cause of cancer death 1,2. The disease is divided into small cell lung cancer (SCLC) and nonsmall cell lung cancer (NSCLC), with ∼8 of 10 being NSCLC.

Currently, 75% of lung cancers are diagnosed late, when tumors may be unresectable and treatment options limited resulting in a 5-year survival rate of only 10% 3,4. No practical way of screening patients at risk of lung cancer exists 5 and detection of lung cancer mostly relies on imaging modalities such as computerized tomography (CT) scans, which is not optimal 6. Thus, improved diagnosis of lung cancer is essential for enhanced survival.

Blood-based molecular biomarkers, which indicate that patients have lung cancer and/or define high-risk patients will have an enormous clinical potential. Unfortunately, the serum biomarkers currently in use for lung cancer are limited to monitoring 7 and no validated molecular biomarkers for the early detection of lung cancer exist despite several investigations carried out with this purpose 8.

Tumor biology includes a complex dynamic interaction between tumor cells and the microenvironment that may lead to a loss of overall tissue homeostasis and promote tumor progression 9. Consequently, biomarkers that reflect such structural changes in the tissue as a whole, and not just tumor cell changes, may have a great potential.

Novel noninvasive molecular cancer biomarkers may be identified by exploiting a technology based on the concept that tumor-associated proteases and tumor signature proteins, potentially combined with specific posttranslational modifications, result in release of unique protein degradation fragments to the circulation 10. The protein fragments contain pathology-specific neoepitopes that may serve as specific cancer biomarker targets that most likely enter the circulation by diffusing into the leaky vasculature found in tumor tissue.

In lung cancer, the intermediate filament (IMF) protein vimentin has been shown to be applicable for clinical pathology 11. In addition, vimentin is regarded as a canonical marker for the epithelial-to-mesenchymal transition (EMT) 12. Vimentin has been identified as a surface and secreted protein 13–15 and may be a target for proteolytic cleavage. Furthermore, vimentin has been shown to be a target for citrullination by peptidylarginine deiminase (PAD) enzymes which are activated during apoptosis and inflammation 16,17.

Extracellular matrix (ECM) remodeling is another key event in cancer 18. The ECM integrating and surrounding malignant tumors is a central part of disease regulation and development. ECM remodeling involves increased and altered local production of ECM proteins (a phenomenon known as fibrosis or desmoplasia) as well as increased and altered ECM degradation by upregulated matrix metalloproteinase (MMP) activity 19. Consequently, this leads to a different composition and quality of the ECM as compared to the normal homeostatic state 20.

In this study, we investigated whether serum biomarkers reflecting ECM and IMF remodeling/dysregulation could be used to differentiate lung cancer patients from healthy controls, other lung pathologies, and other cancer types. In detail, we measured specific protein fingerprints reflecting MMP-degradation and citrullination of vimentin, MMP-degradation of type I collagen, the main component of the structural interstitial ECM, and MMP-degradation of elastin which provides elasticity to the lung tissue.

## Material and Methods

### Patient samples

After informed consent and approval by appropriate Institutional Review Board/Independent Ethical Committee, serum was collected from patients and age-matched healthy controls with no symptomatic or chronic disease. Patient samples were obtained from the commercial vendors Asterand (Detroit, MI) and Proteogenex (Culver City, CA) and the healthy controls were a pool of samples from Asterand and another study population 21. According to Danish law, it is not required to get ethical approval when measuring biochemical markers in previously collected samples; hence, there was no additional ethical approval for this particular study. Demographics and clinical profiles are shown in Table 1. Samples were all collected, processed, and stored in a similar fashion until analyzed, and all analyses were performed blindly. Patient samples were collected prior to surgery. Tumor stage and histological type were classified according to the criteria of the American Joint Committee on Cancer (AJCC) and International Union against Cancer (IUCC).

**Table 1:** Patient demographics and clinical profiles

			Tumor stage (*n*)				Smoking status			
			Tumor stage (*n*)				Smoking status			
					
Group	No. of patients	Gender, % females	I	II	III	IV	Never	Ever	Unknown	Age, mean ± SD (range)
Lung cancer, all	40	25	13	12	12	3	3	35	2	59 ± 10 (46–82)
SCLC	8	25	2	1	4	1	–	7	1	61 ± 12 (46–82)
NSCLC	32	25	11	11	8	2	3	28	1	60 ± 9 (46–80)
Adenocarcinoma	16	37.5	4	6	6	–	–	15	1	57 ± 10 (46–80)
Squamous cell carcinoma	16	12.5	7	5	2	2	3	13	–	63 ± 6 (53–73)
Gastrointestinal cancer (*adenocarcinoma*)	25	52	4	10	10	1	5	6	14	62 ± 11 (38–81)
Prostate cancer (*adenocarcinoma*)	14	0	1	13	–	–	1	6	7	64 ± 6 (52–72)
Malignant melanoma of the skin	7	43	1	5	1	–	–	1	6	46 ± 14 (30–64)
COPD^1^ (moderate/severe)	13	77	–	–	–	–	12	1	–	72 ± 4 (67–80)
IPF (FEV 63–68%)	10	20	–	–	–	–	9	1	–	74 ± 5 (67–83)
Healthy controls	33	52	–	–	–	–	–	–	33	61 ± 11 (43–78)

COPD, Chronic Obstructive Pulmonary Disease; FEV, Forced Expiratory Volume (in spirometer); IPF, Idiopathic Pulmonary Fibrosis; NSCLC, nonsmall cell lung cancer; SCLC, small cell lung cancer.

1 Hazard occupation.

### ELISA measurements and procedure

The levels of MMP-degraded type I collagen (C1M) 22, MMP-degraded elastin (ELM) 23, and MMP-degraded citrullinated vimentin (VICM) 24 were assessed in serum samples using well characterized competitive enzyme-linked immunosorbent assay (ELISAs). The targets were identified from in vitro and ex vivo studies and by use of mass spectrometry and all the biomarker assays are technically validated (see each reference for details).

In brief, the assays were performed by dissolving a biotinylated synthetic target peptide in an optimized assay buffer that was added to a 96-well streptavidin coated plate, which then incubated for 30 min at 20°C. The plate was washed five times in wash-buffer (20 mmol/L Tris, 50 mmol/L NaCl, pH 7.2) prior to addition of 20 *μ*L target peptide calibrator or sample and 100 *μ*L of a horseradish peroxidase-conjugated monoclonal antibody rose against the target peptide-sequence of interest. The plate incubated for 1–2 h at 20°C or overnight at 4°C, depending on the individual assay. The plate was washed five times in wash-buffer and finally 100 *μ*L tetramethylbenzinidine (Kem-En-Tec cat.438OH) was added and the plate was incubated for 15 min at 20°C in dark. The reaction was stopped by adding 100 *μ*L of stopping solution (1% H_2_SO_4_) and the OD_450–650nm_ was measured.

### Statistical analysis

The levels of the individual biomarkers in serum samples were compared using analysis of variance (ANOVA) test with Dunnetts test to adjust for multiple comparisons or an unpaired *t*-test on Log10 transformed data. Data are presented as Tukey box plots. The area under the receiver operating characteristics (AUROC) was calculated for each biomarker and for the biomarkers combined. A diagnostic decision tree was generated from specific cutoff values and analyzed using Fisher's exact probability test and the chi-square test. Statistical analyses were performed using MedCalc Statistical Software v.12 (MedCalc Software, Ostend, Belgium) and GraphPad Prism v.6 (GraphPad Software, La Jolla, CA). Results were considered statistically significant if *P* < 0.05.

## Results

### Extracellular matrix degradation and citrullinated vimentin degradation in serum from patients with various cancers and controls—lung cancer stands out

**Figure 1:**
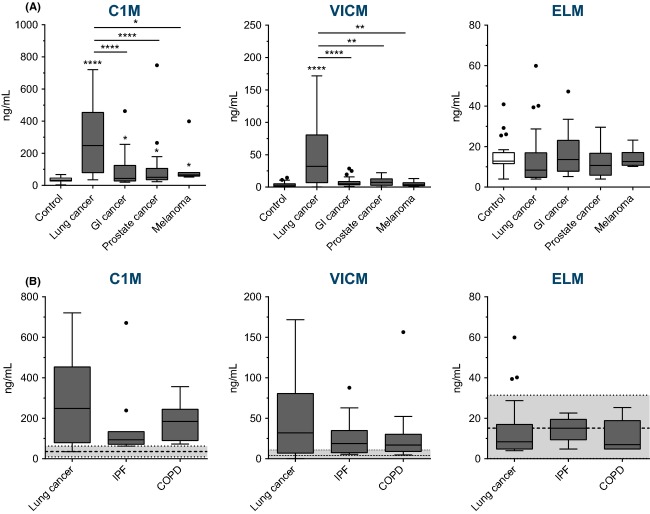
Levels of MMP-generated fragments of type I collagen (C1M), citrullinated vimentin (VICM), and elastin (ELM) in serum from: (A) patients with lung cancer (*n* = 40), gastrointestinal (GI) cancer (*n* = 25), prostate cancer (*n* = 14), malignant melanoma (*n* = 7), and healthy controls (*n* = 33); and (B) patients with lung cancer (*n* = 40), chronic obstructive pulmonary disease (COPD) (*n* = 13), idiopathic pulmonary fibrosis (IPF) (*n* = 10). In (B), healthy controls (*n* = 33) are illustrated by the dotted line (mean) and the gray area (±2 SD). Controls were compared to each cancer type and lung cancer compared to the other cancer types and lung pathologies by Dunnetts ANOVA multiple comparisons test on Log10 transformed data. Significance levels: *P < 0.05, ** *P* < 0.01, ****P < 0.0001.

The levels of specific C1M, VICM, and ELM were measured in serum from various cancer patients and healthy controls (Fig.1A).

C1M was significantly elevated in all cancer types. The highest levels were reached in lung cancer patients with an average increase of eightfold (*P* < 0.0001) when compared to controls, and with the other cancer types having a two- to threefold average increase (*P* < 0.05) as compared to controls. Furthermore, lung cancer patients had significantly higher levels of C1M when compared to the other cancer types (*P* < 0.05–0.0001).

Together, these findings indicate that altered collagen turnover is ongoing in cancer, with lung cancer having the highest levels of type I collagen degradation. This reflects that alterations in the generation and degradation of collagens and release of specific protein fragments to the circulation are a pathological feature of cancer, especially lung cancer.

For VICM, the levels were significantly elevated only in lung cancer. The average increase was approximately tenfold when compared to either healthy controls (*P* < 0.0001) or the three other cancer types (*P* < 0.01–0.0001). This finding indicates that citrullination and MMP-degradation of (secreted) vimentin and the release to the circulation is associated with lung cancer only and not other cancer types.

Finally, no difference in the levels of ELM could be detected, indicating that alterations in the generation and release of these protein fragments to the circulation are not a pathological feature of the cancers analyzed in this study.

Next, the AUROC was calculated as a measure of the diagnostic power of the biomarkers individually and combined. This was done for all lung cancer patients versus the healthy controls and for all lung cancers versus the other cancer types analyzed in this study combined (GI, prostate, and melanoma cancer) (Table 2). The results shows that when analyzing all lung cancer patients versus healthy controls the diagnostic power of C1M, and VICM individually were highly significant with an AUROC of 0.97 and 0.85, respectively (*P* < 0.0001). The diagnostic power of ELM was somewhat poorer with an AUROC of 0.67 (*P* = 0.013). Interestingly, when combining C1M and VICM, a diagnostic power of 0.98 (*P* < 0.0001) was achieved, indicating that a complete discrimination between healthy controls and lung cancer patients is obtained. When the lung cancer patients were compared to the other cancers combined, the markers showed promising discriminative power as well. Here, C1M and VICM individually as well as C1M and VICM combined were highly significant with AUROCs of 0.81, 0.79, and 0.83, respectively (*P* < 0.001).

**Table 2:** Diagnostic power of the biomarkers for all lung cancers and for the nonsmall cell lung cancer subtype (NSCLC) calculated as the area under the receiver operating characteristic (AUROC)

Biomarker	AUROC	95% CI	Specificity %	Sensitivity %	*P*-value
*Lung cancers (all)*					
Versus healthy controls					
C1M	0.97	0.895–0.994	94	90	<0.0001
VICM	0.85	0.752–0.926	100	65	<0.0001
ELM	0.67	0.543–0.784	84	63	0.013
C1M + VICM	0.98	0.956–1.0	100	90	<0.0001
Versus other cancers					
C1M	0.81	0.714–0.891	75	75	<0.0001
VICM	0.79	0.688–0.872	93	63	<0.0001
ELM	0.65	0.538–0.751	98	35	0.0157
C1M + VICM	0.83	0.736–0.924	91	65	<0.0001
Versus other lung pathologies					
C1M	0.64	0.511–0.758	96	45	0.046
VICM	0.59	0.455–0.709	78	56	0.239
ELM	0.58	0.450–0.705	100	23	0.265
C1M + VICM	0.66	0.522–0.795	96	45	0.0225
*NSCLC*					
Versus all others					
C1M	0.83	0.751–0.913	72	81	<0.0001
VICM	0.84	0.768–0.920	86	69	<0.0001
ELM	0.65	0.518–0.776	85	47	0.0254
C1M + VICM	0.88	0.823–0.940	71	94	<0.0001
Versus other lung pathologies					
C1M	0.69	0.545–0.803	96	53	0.013
VICM	0.66	0.515–0.778	78	63	0.040
ELM	0.59	0.450–0.722	100	25	0.237
C1M + VICM	0.72	0.586–0.835	87	56	0.001

### Specificity of the markers for lung cancer as compared to other lung pathologies

We wanted to address in more detail if the markers were specific for lung cancer as compared to other lung pathologies such as idiopathic pulmonary fibrosis (IPF) and chronic obstructive pulmonary fibrosis (COPD). For all the markers tested, no statistical difference could be detected between lung cancer and either COPD and IPF (Fig.1B). However, clearly a trend toward increasing levels and a wider distribution of C1M and VICM was observed in lung cancer. Furthermore, when calculating the AUROC with respect to lung cancer versus other lung pathologies (i.e., IPF and COPD combined) (Table 2), the discrimination turned out significant for C1M alone with an AUROC of 0.64 (*P* < 0.05), and for the combination of C1M and VICM with an AUROC of 0.66 (*P* < 0.05). This suggests that the markers may have an ability to distinguish between the two groups of lung diseases although with relatively poor accuracy. Together the findings indicate that the markers reflect lung pathology tissue turnover-dependent mechanisms in general, and that these mechanisms may be more pronounced in lung cancer (or a subgroup of lung cancers) as compared to the other lung pathologies (IPF + COPD) tested.

### The biomarker expression profiles according to lung cancer subtype and tumor stage

**Figure 2:**
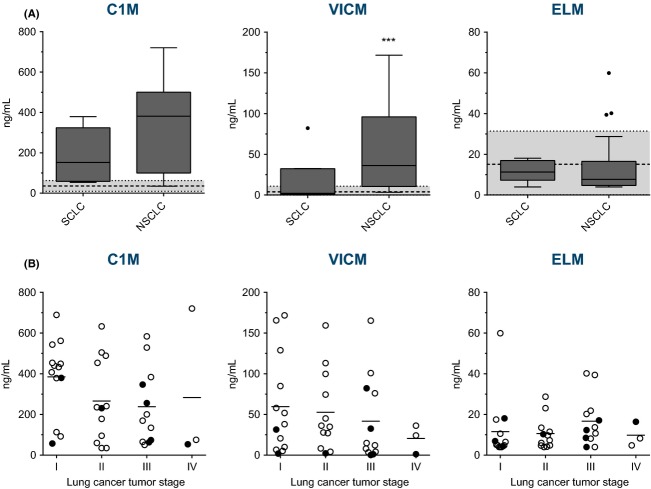
Levels of MMP-generated fragments of type I collagen (C1M), citrullinated vimentin (VICM), and elastin (ELM) in serum from lung cancer patients: (A) divided into small cell lung cancer (SCLC) (*n* = 8) and nonsmall cell lung cancer (NSCLC) (*n* = 32) and with the mean of the healthy controls (*n* = 33) illustrated by the dotted line ±2 SD (gray area); and (B) divided into stage of the disease for both SCLC (•) and NSCLC (○) and with the mean illustrated by a horizontal line. Groups were compared using an unpaired *t*-test (A) and Dunnetts ANOVA multiple comparisons test (B) on Log10 transformed data. Significance levels: ***P < 0.001.

Lung cancer is divided into two major subtypes which are important in the clinical setting: SCLC and NSCLC. As there is a trend toward increasing levels and a wider distribution of especially C1M and VICM levels in serum from the lung cancer population, the difference in biomarker levels between the lung cancer subtypes was assessed. When comparing SCLC and NSCLC we found that VICM (*P* < 0.001) was significantly elevated in NSCLC as compared to SCLC with an average increase of threefold (Fig.2A). Although no significant difference could be detected with C1M (*P* = 0.142) on average a twofold increase was seen in NSCLC as compared to SCLC. For ELM no difference was detected. Overall, these findings indicate that the tissue alterations assessed in this study is more pronounced in NSCLC as compared to SCLC. As shown in Table 2 when analyzing NSCLC versus all other patients plus the healthy controls the diagnostic power of C1M, and VICM individually were significant with an AUROC of 0.83 and 0.84, respectively (*P* < 0.0001) and when combined (C1M + VICM) with an AUROC of 0.88 (*P* < 0.0001). Furthermore, NSCLC could be discriminated from other lung pathologies; when combining C1M and VICM an AUROC of 0.72 was obtained (*P* < 0.001).

The tumor stage of disease is another important clinical tool in lung cancer cases. As illustrated in Figure2B, no significant difference was detected between the different tumor stages. Thus, an association between tumor stage and level of biomarkers could not be detected in this study. Furthermore, as the markers were elevated in all stages of the disease it may be suggested that they have potential as markers of early lung cancer.

### Diagnostic decision tree for predicting the likelihood of having lung cancer

**Figure 3:**
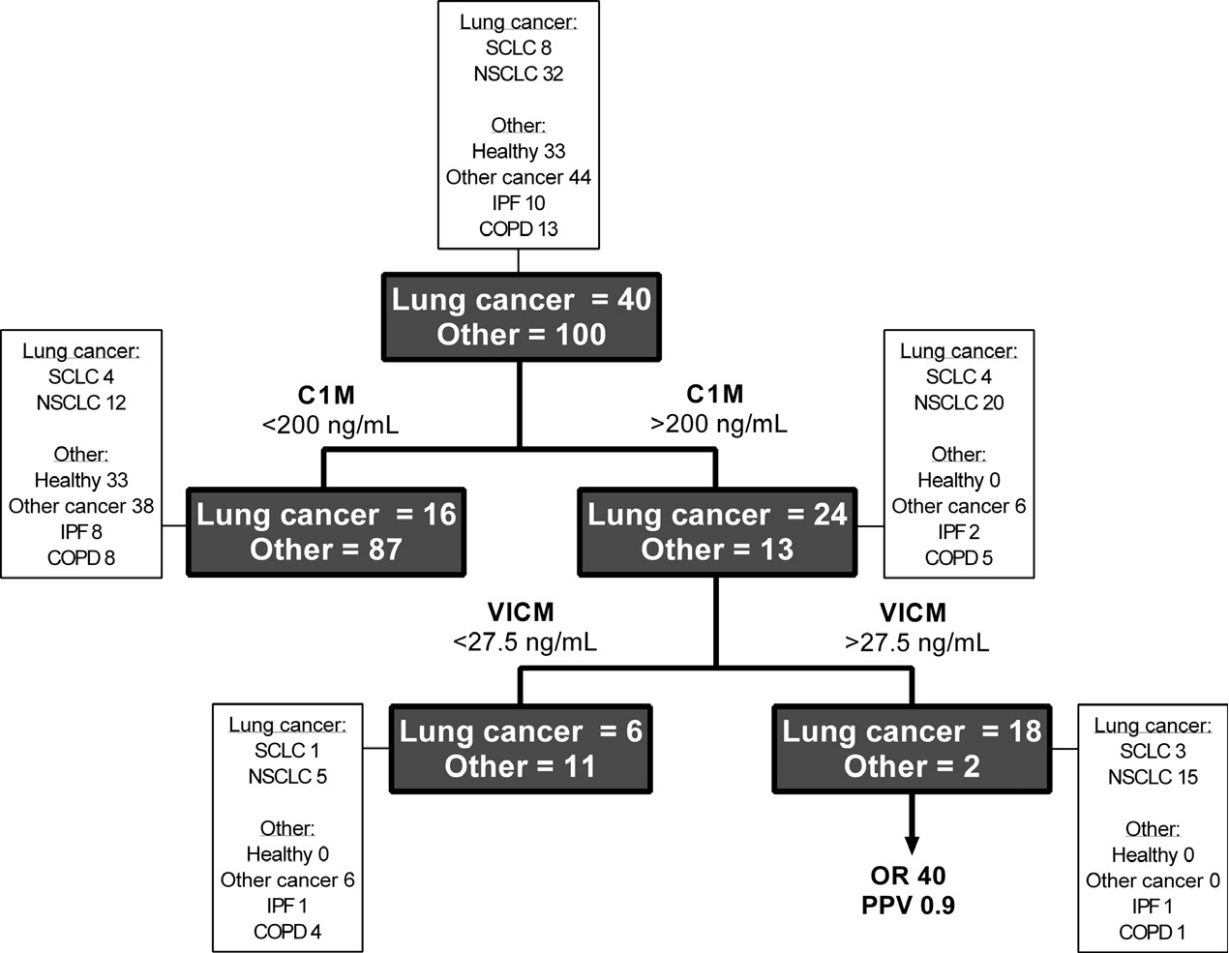
Diagnostic decision tree for identifying lung cancer patients in the study current cohort. The tree (dark gray boxes) was constructed based on defined biomarker cutoff values for C1M (200 ng/mL) and VICM (27.5 ng/mL) that were determined by calculating the mean of the biomarker levels from all the samples measured (*n* = 140). Details on patient types in each of the tree branches are shown in the white boxes. OR, Odds Ratio for having lung cancer; PPV, positive predictive value of the test. Statistical details are shown in Table 3.

In an attempt to produce a model for predicting the likelihood of having lung cancer, a diagnostic decision tree was constructed. The initial split for being lung cancer positive was made at C1M >200 ng/mL and followed by VICM >27.5 ng/mL. The patient distribution and statistics according to this algorithm are illustrated in Figure3 and in Table 3. With a sensitivity of 45% and specificity of 98%, the two markers of C1M and VICM successfully identified a subpopulation of patients that with 90% certainty (PPV 0.9) are positive for having lung cancer. This corresponds to 45% (18/40) of all lung cancer patient analyzed or 13% (18/140) of the tested population. This subgroup of patients with a high level of C1M and VICM as defined by the cutoff values described above had an odds ratio of 40 (95% CI 8.7–186, *P* < 0.0001) suggesting that when levels of C1M >200 ng/mL and VICM >27.5 ng/mL are detected in a patients' blood sample, the probability of having lung cancer is 40 times that of not having lung cancer.

**Table 3:** Probability of having lung cancer when C1M is >200 ng/mL and when C1M >200 ng/mL + VICM >27.5 ng/mL. Statistical details are calculated from the diagnostic decision tree

Biomarker	OR	95% CI	*P*-value	Sensitivity (%)	Specificity (%)	PPV	NPV	LHR
C1M	10	4.2–24	<0.0001	60	87	0.65	0.85	4.6
C1M + VICM	40	8.7–186	<0.0001	45	98	0.9	0.83	22.5

LHR, likelihood ratio; OR, odds ratio; PPV/NPV, positive/negative predictive value.

## Discussion

We measured degradation products in serum of two ECM proteins: type I collagen (C1M) and elastin (ELM). Furthermore, we measured degradation products of citrullinated vimentin (VICM). The biomarker targets used in this study were identified from in vitro and ex vivo studies and by use of mass spectrometry. The background for choosing C1M and ELM was based on previous findings from fibrosis-related diseases of the lung (IPF and COPD, 25). VICM was chosen based on the role of vimentin as a marker of EMT. VICM has also been associated with fibrosis 24 and we speculated that it may be linked to cancer as well.

C1M and VICM were significantly elevated in lung cancer as compared to controls and other cancer types. A trend was detected when comparing lung cancer with other lung pathologies. No difference could be seen for ELM. When separating the lung cancer subtypes SCLC and NSCLC, it was clear that the higher levels in the markers could be associated with NSCLC and for VICM the level in NSCLC patients was significantly higher than any other group.

Although this study is limited by a small sample size and lack of patient information, clearly, there seems to be potential for the biomarkers presented to be applied in the field of lung cancer: C1M and VICM successfully identified a subpopulation of patients with lung cancer (Fig.3). In addition, the markers were elevated in all stages of the disease indicating that they may have potential as markers of early disease.

This study is the first to indicate that the release of high levels of citrullinated fragments of vimentin (VICM) to the circulation may be a specific signal for lung disease. This finding is supported by the association between citrullinations and smoking and induction of inflammation 26 and in line with cigarette smoke exposure as the major risk factor for lung cancer. Unpublished data from our group clearly indicate that none of the biomarkers used in this study are influenced by smoking.

Type I collagen is present in most organs, but increased in the stroma of neoplastic tissue leading to desmoplasia 27,28. Furthermore, it is well known that an increased production of MMP is associated with cancer. This combination may lead to a high degree of type I collagen degradation (C1M) in concordance with our findings. C1M together with degradation products of other collagens have recently been found elevated in patients with pancreatic ductal adenocarcinomas, a cancer characterized by severe desmoplasia 29 and elevated levels of circulating fragments of type I collagen have been found to be predictors of poor outcome in lung cancer 30,31 as well as found elevated in several other cancers such as for instance head and neck cancer 32 and ovarian cancer 33,34. C1M has also previously been found elevated in COPD and IPF patients 25, similar to our findings, indicating that C1M may be a biomarker of several pulmonary diseases and suggesting that lessons may be learned from research in lung fibrotic diseases such as COPD 35 and IPF 36, which may benefit development of novel biomarkers for lung cancer (and vice versa). Pulmonary fibrosis and lung cancer share many pathological similarities; as a trend that is observed with lung cancer having higher levels of C1M and VICM as compared to pulmonary fibrosis, one might speculate that a higher degree of tissue turnover (dynamic change) is observed in lung cancer tissue. This of course needs validation in a future study.

Both COPD and IPF have been linked to lung cancer and whereas studies indicate that COPD is a risk factor for lung cancer and that COPD patients might have underlying lung cancer 37,38, results are somewhat more contradictory for IPF 39. Still, IPF have been described as a prognostic factor in NSCLC 40 and in a study by Nagai et al., lung cancer was found to be present in one-third of the IPF patients analyzed 41. We speculate that it might be possible that the two (10%) false positive lung cancer patients from the diagnostic decision tree (i.e., one IPF patient and one COPD patient), may have underlying undiagnosed lung cancer.

The mechanism behind the degradation and modification of the highly complex mixture of ECM and IMF proteins is still not fully understood, however, studies indicate that the composition of MMPs and thereby the degradation products may differ between lung cancer subtypes 42–44. As MMPs display diverse and sometimes opposite effects depending on tissue localization, cellular source, stage, and cancer subtype 45,46 together this may contribute, in a complex manner additional to the complexity of the ECM and IMF status and to the differences observed between patient subtypes in this study.

Several serum-based lung cancer biomarkers are currently available in the clinical setting; however, their use is of limited capacity and not recommended 8. Many proteins have been studied as potential serum-based biomarkers for lung cancer. For instance, a panel of serum proteins (carcinoembryonic antigen, retinol-binding protein, *α*1-antitrypsin, and squamous cell carcinoma antigen) has been shown to be of promising value in the clinical setting 47. All markers are MMP driven, and consequently they may reflect the activation or inhibition of specific MMPs in vivo which are needed in designing anti-MMP treatment strategies 48. Furthermore, the role of citrullinations and PAD enzymes in cancer has made it relevant to test PAD inhibition as a treatment strategy 49,50.

In conclusion, this is the first study to test biomarkers specifically reflecting ECM and IMF remodeling as diagnostic tools for identifying lung cancer patients. We speculate that noninvasive serum biomarkers as described, may aid in the diagnosis and characterization of patients with lung cancer in the future.
